# Severe Burns of the Genital Area After Laser Hair Removal: A Case Report

**DOI:** 10.7759/cureus.47429

**Published:** 2023-10-21

**Authors:** Ilina Braynova, Pavel Timonov, Antoaneta Fasova, Alexandar Alexandrov

**Affiliations:** 1 Forensic Medicine and Deontology, Medical University, Sofia, Sofia, BGR; 2 Forensic Medicine, St. George University Hospital, Plovdiv, BGR; 3 Forensic Medicine and Deontology, Medical University of Plovdiv, Plovdiv, BGR; 4 Anatomy, Medical University of Plovdiv, Plovdiv, BGR

**Keywords:** scar formation, complications, laser hair removal, unwanted hair removal, burns

## Abstract

Burn injuries are among the most commonly observed complications of laser hair removal. Here, we present a case, in which severe massive burns were caused in the genital and perineal areas during such a procedure. The consequent scar formation led not only to negative aesthetic effects but also affected the physical and psychological health of the patient.

## Introduction

Injuries caused by exposure to high temperatures, including hot gases and objects radiating heat energy, are referred as burns. Meanwhile, injuries caused by hot liquids are called scalds [1]. The incidence of burns as intentional assaults varies between 3% and 10% from all hospitalized patients [2]. The significance of morbidity and mortality rates due to burns worldwide remains high. The damage of the skin integrity leads to increased risk of infections, which in some cases is complicated with sepsis [3]. The reason for reporting our case is to pay attention to the long-term impairment of health, even though it was not a fatal burning case. 

Laser and light pulse epilation have emerged as leading treatment options for long-term unwanted-hair removal [4]. Hair removal with laser devices (alexandrite, diode, neodymium-doped yttrium aluminum garnet (Nd:YAG), and ruby lasers) and intense pulsed light are commonly used even though the long-term effects are uncertain [5]. Although light pulse epilation and laser epilation have a balancing act between the maximal therapeutic effect and minimal side effect risk, there are complications connected with the skin integrity described. Some of the possible complications of laser and intense pulsed light hair removal are burns. Radmanesh et al. (2018) pointed out that leukotrichia, paradoxical hypertrichosis, and folliculitis are also commonly observed complications of photoepilation. Riml et al. (2013) reported a case of second-degree burn in a tattooed skin area after laser epilation [6]. According to Bayle et al. (2015), laser hair removal in France is restricted by law to medical use and is also a subject of legal and economic controversy. The operator's liability may be engaged, especially in cases with complication development and those that include forensic examinations and legal measures on the provider of the procedure [7]. According to the most comparative research studies, the effectiveness of intense pulsed light and laser hair removal is similar [8,9,10].

## Case presentation

**Figure 1:**
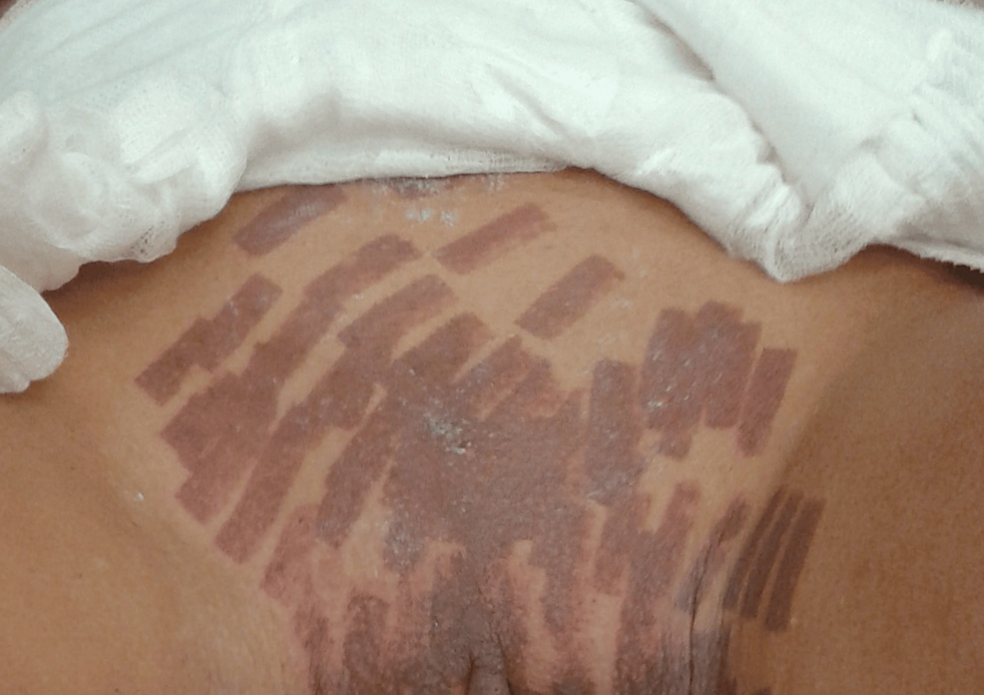
View of the burns a day after the laser hair removal procedure and medical treatment.

We present a case of a 32-year-old woman that had severe burns during a laser hair removal procedure in the genital area and perineum. During the whole procedure, she felt severe pain and burns, and she kept informing the operator performing the procedure. Nevertheless, she was told that it was just normal pain, and if she wanted the effect, she need to tolerate the pain. At the end of the procedure, she continued to feel severe pain, so she went to the emergency room. It was then established that she had severe, multiple burns of the genital perigenital and perineal areas, which were of the second degree (Figure 1).

**Figure 2:**
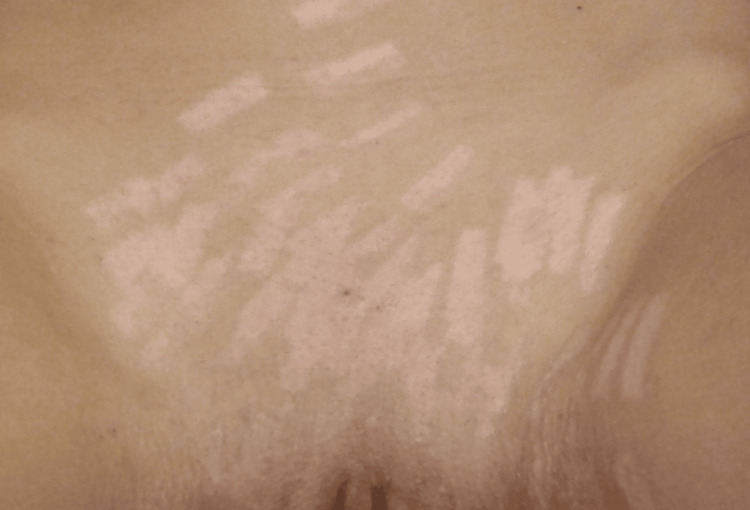
View of the scars formed after the healing process finished (five weeks after the laser hair removal procedure).

She was treated, medically and surgically. The necrotic tissues were removed. The woman was examined in the forensic department the day after she got burnt. The forensic examiner established findings that fully corresponded to the clinical record of the patient: the superficial layer of the skin was brownish, and there were areas of treated blisters and removal of necrotic tissues. The patient was advised to come back again after at least four weeks for a second examination and assessment of the severity of the injuries and the scar formation. It was also necessary to give a conclusion of the severity of scarring that would permanently remain. After the second examination, five weeks after the burning, the presence of massive multiple hypopigmented areas, whitish in color, was registered (Figure 2). The hypopigmented areas, even though treated properly, would permanently remain and be visible. 

## Discussion

Therapeutic lasers can be used for five main medical indications: vascular coagulation, pigment ablation, facial rejuvenation, tissue cutting or ablation, and hair removal. Unwanted-hair removal with laser is becoming a more common and popular preferred method in aesthetic medicine. The possible complications of laser hair removal are burns, leukotrichia, paradoxical hypertrichosis, and folliculitis. Burns are classified according to the depth of the skin injury. In cases of deep second-degree (2B) and higher-grade burns where the epidermis and skin appendages are destroyed, healing would only take place with severe scarring [11]. As in all burn cases, when the depth of the thermal injury is deep second or higher grade, the healing process, even with medical treatment, includes scar formation. Once formed, the scars would remain for life. They might lead not only to anatomical and aesthetical changes but might also cause permanent dysfunctions. Despite the fact that acute burn care is improving, up to 70% of patients develop hypertrophic scars, which can result in functional and psychosocial negative effects [12]. If the burns are deeper than the dermis, the healing process includes scar formation, which in some cases might lead to not only anatomical but also functional changes. The description of scars is based on appearance, such as color (red, purple, and blanching), texture, thickness, raised/depressed, elasticity, and functional effects [13]. Psychological distress and the connecting challenges with community reentry also need to be paid attention [13].

In the presented case, the burns of the skin in the genital and perineal areas were of second grade. Two forensic examinations were performed - first, on the day after the procedure and, second, five weeks after it. During the second examination, the formation of multiple whitish hypopigmented areas was registered, which would remain for a relevant long period of time. Another examination was recommended to the patient. The aesthetic center of the laser epilation procedure and the person operating the laser have legal responsibilities. Even though the woman complained that she felt burns and intensive pains, the procedure was not stopped and reassumed. In this case, the laser operator, who ought to check if there was a problem with the procedure, should be held responsible.

## Conclusions

Although laser hair removal is preferred for its long-lasting effect, it is connected with serious medical risks. The most often observed complication is burns. Such complications should and may be prevented. This is a medical and legal responsibility of the medical provider of the epilation procedure and personally of the laser operator. The forensic expert evaluated the present condition of the patient and gave basis for the investigation and legal conclusion for the legal consequences in the case.

## References

[1] Yakupu A, Zhang J, Dong W, Song F, Dong J, Lu S (2022). The epidemiological characteristic and trends of burns globally. BMC Public Health.

[2] Peck MD (2012). Epidemiology of burns throughout the world. Part II: intentional burns in adults. Burns.

[3] Souto EB, Ribeiro AF, Ferreira MI (2020). New nanotechnologies for the treatment and repair of skin burns infections. Int J Mol Sci.

[4] Gan SD, Graber EM (2013). Laser hair removal: a review. Dermatol Surg.

[5] Haedersdal M, Gøtzsche PC (2006). Laser and photoepilation for unwanted hair growth. Cochrane Database Syst Rev.

[6] Riml S, Larcher L, Grohmann M, Kompatscher P (2013). Second-degree burn within a tattoo after intense-pulsed-light epilation. Photodermatol Photoimmunol Photomed.

[7] Bayle P, Saval F, Rougé D, Telmon N (2015). Complications after laser hair removal: the standpoint of a dermatological legal expert regarding liability [Article in French]. Ann Dermatol Venereol.

[8] Babilas P, Schreml S, Szeimies RM, Landthaler M (2010). Intense pulsed light (IPL): a review. Lasers Surg Med.

[9] Prohaska J, Hohman M (2023). Laser Complications. StatPearls [Internet].

[10] Radmanesh M, Azar-Beig M, Abtahian A, Naderi AH (2008). Burning, paradoxical hypertrichosis, leukotrichia and folliculitis are four major complications of intense pulsed light hair removal therapy. J Dermatolog Treat.

[11] Daigeler A, Kapalschinski N, Lehnhardt M (2015). Therapy of burn injuries [Article in German]. Chirurg.

[12] Kamolz LP, Hecker A (2023). Molecular mechanisms related to burns, burn wound healing and scarring. Int J Mol Sci.

[13] Obaidi N, Keenan C, Chan RK (2023). Burn scar management and reconstructive surgery. Surg Clin North Am.

